# Supplementary material for: The Human Monocyte—A Circulating Sensor of Infection and a Potent and Rapid Inducer of Inflammation
Source: Int J Mol Sci. 2022 Mar 31;23(7):3890. doi: 10.3390/ijms23073890 (PMC8999117; doi:10.3390/ijms23073890)
Supplement: Supplementary file 1 [file ijms-23-03890-s001.zip › ijms-1654882-supplementary.pdf]

Supplementary Tables

Table S1 Lysozyme, Actin and MHC

The five diferent donors are listed as 1-5. Donors 1-4 were treated with 1 ug/ml of *E. coli* LPS and donor 5 with 200 ng/ml of IFN-γ. Donor 1 is a male age 51, donor 2 a male age 43, donor 3 a female age 47, donor 4 a male age 28 and donor 5 a female age 61.

Gene	0	4h	24h	48h	4h+LPS (IFN-γ)	24h+LPS (IFN-γ)	48h+LPS (IFN-γ)
Lysozyme 1.	27394	-	-	-	5200	480	2658
2.	29951	-	-	-	1990	11457	6628
3.	25335	7417	16476	21289	3130	245	2914
4.	16400	4836	5469	6410	3941	419	1740
5.IFN	11290	4227	7457	12834	4248	16429	22705
Actin-b 1.	19693	-	-	-	6700	8574	7094
2.	21775	-	-	-	13102	17563	13373
3.	14866	6943	11192	11799	5027	9130	13934
4.	23328	29919	19278	31241	8769	11644	22776
5.IFN	32168	19263	82706	20846	26870	36242	31642
HLA-DRA 1.	5490	-	-	-	5705	733	654
2.	7224	-	-	-	4227	3819	921
3.	934	1337	1579	2739	1145	52	108
4.	6558	6151	5958	8558	8578	858	722
5.IFN	3645	2592	2964	7227	2262	11944	11375
HLA-DRB1 1.	3023	-	-	-	1748	421	528
2.	26	-	-	-	1	8	3
3.	1820	1897	3980	6568	1227	108	327
4.	21	184	15	16	7	0.7	10
5.IFN	2188	2126	3042	5800	2523	7713	6935
HLA-DPA1 1.	2375	-	-	-	649	147	305
2.	4872	-	-	-	377	2156	846
3.	1656	1332	2660	5358	548	28	55
4	5268	3616	3802	5241	2065	263	302
5.IFN	4099	2807	4661	7697	6769	16952	16471
HLA-DPB1 1.	1029	-	-	-	360	68	83
2.	2162	-	-	-	89	738	245
3.	551	665	1122	2553	186	6	19
4.	1478	1423	1359	2090	799	76	104
5.IFN	1506	978	2002	4441	2451	5613	5399
HLA-DPB2 1.	0	-	-	-	0.3	0	4
2.	0.4	-	-	-	0	0.3	0
3.	0	0	0	0	0	0.7	0.2
4.	0	0	0	0	0	0.1	0
5.IFN	0.1	0	0	0	0.2	0	0
HLA-DQA1 1.	103	-	-	-	186	154	93
2.	6	-	-	-	0.5	3	2
3.	86	85	309	735	94	6	16
4.	9	39	3	8	3	0.7	6
5.IFN	378	285	975	1596	1163	3237	2473

**HLA-DQA2**	**1.**	**0**	**-**	**-**	**-**	**0**	**0.2**	**0.3**
	**2.**	**0**	**-**	**-**	**-**	**0.3**	**0.5**	**0.2**
	**3.**	**0**	**0**	**0**	**0**	**0**	**0.2**	**0**
	**4.**	**0.8**	**0.2**	**0.8**	**0**	**0.4**	**0.1**	**0.5**
	**5.IFN**	**0**	**0**	**0**	**0.3**	**0**	**0.3**	**0.1**
**HLA-DQB2**	**1.**	**0**	**-**	**-**	**-**	**0**	**0**	**0**
	**2.**	**0**	**-**	**-**	**-**	**0**	**0**	**0.2**
	**3.**	**0**	**0**	**0**	**0**	**0**	**0**	**0**
	**4.**	**0.3**	**0**	**0**	**0**	**0**	**0**	**0**
	**5.IFN**	**0**	**0**	**0.4**	**0.2**	**0.8**	**0.8**	**0.3**
**HLA-DMA**	**1.**	**254**	**-**	**-**	**-**	**70**	**23**	**29**
	**2.**	**308**	**-**	**-**	**-**	**54**	**244**	**122**
	**3.**	**290**	**177**	**596**	**778**	**71**	**4.9**	**14**
	**4.**	**412**	**318**	**463**	**611**	**146**	**26**	**42**
	**5.IFN**	**415**	**203**	**761**	**792**	**374**	**1153**	**918**
**HLA-DMB**	**1.**	**0.3**	**-**	**-**	**-**	**0.1**	**0**	**0.2**
	**2.**	**35**	**-**	**-**	**-**	**5**	**18**	**8**
	**3.**	**0.3**	**0**	**0**	**0.3**	**0**	**0**	**0.2**
	**4.**	**0.1**	**0.5**	**0.2**	**0**	**0**	**0**	**0**
	**5.IFN**	**0**	**0**	**0.4**	**0**	**0**	**0.1**	**0**
**HLA-DOA**	**1.**	**12**	**-**	**-**	**-**	**2**	**3**	**4**
	**2**	**13**	**-**	**-**	**-**	**2**	**3**	**3**
	**3.**	**2**	**0**	**4**	**7**	**0.6**	**0**	**0.2**
	**4.**	**7**	**3**	**3**	**6**	**2**	**3**	**0.7**
	**5.IFN**	**12**	**5**	**10**	**36**	**14**	**34**	**59**
**HLA-DOB**	**1.**	**0.1**	**-**	**-**	**-**	**0.4**	**0.2**	**1**
	**2.**	**0.5**	**-**	**-**	**-**	**5**	**5**	**11**
	**3.**	**0.2**	**0**	**0.2**	**0.5**	**0.6**	**0**	**0.6**
	**4.**	**2**	**0**	**0.5**	**0.1**	**0.5**	**7**	**4**
	**5.IFN**	**0.3**	**0.2**	**0**	**0.6**	**0.2**	**0.9**	**0.2**
**CD74**	**1.**	**1088**	**-**	**-**	**-**	**378**	**99**	**116**
	**2.**	**673**	**-**	**-**	**-**	**90**	**758**	**366**
**(Inv. chain)**	**3.**	**62**	**47**	**75**	**163**	**20**	**0.9**	**11**
	**4.**	**419**	**353**	**448**	**733**	**420**	**36**	**52**
	**5.IFN**	**840**	**295**	**459**	**2030**	**171**	**2359**	**4603**
**CIITA**	**1.**	**73**	**-**	**-**	**-**	**7**	**24**	**39**
	**2.**	**94**	**-**	**-**	**-**	**39**	**150**	**84**
**(Transcr.**	**3.**	**238**	**290**	**1508**	**884**	**29**	**13**	**30**
**factor)**	**4.**	**599**	**210**	**275**	**482**	**106**	**51**	**60**
	**5.IFN**	**245**	**183**	**1859**	**828**	**2766**	**1366**	**1172**
**HLA-A**	**1.**	**1548**	**-**	**-**	**-**	**1594**	**1466**	**1165**
	**2.**	**1186**	**-**	**-**	**-**	**481**	**328**	**276**
	**3.**	**420**	**680**	**477**	**704**	**560**	**350**	**298**
	**4.**	**383**	**497**	**395**	**147**	**576**	**211**	**338**
	**5.IFN**	**1755**	**1598**	**5805**	**894**	**3813**	**4053**	**4797**
**HLA-B**	**1.**	**6**	**-**	**-**	**-**	**7**	**5**	**4**
	**2.**	**8**	**-**	**-**	**-**	**3**	**2**	**2**
	**3.**	**1780**	**1715**	**893**	**1164**	**1599**	**925**	**717**
	**4.**	**39**	**196**	**17**	**8**	**31**	**21**	**48**
	**5.IFN**	**1329**	**3005**	**2083**	**1240**	**2452**	**4958**	**4207**

HLA-C	1.	2979	-	-	-	2347	1425	1044
	2.	1265	-	-	-	907	701	779
	3.	403	367	143	144	303	191	125
	4.	5	14	5	0.4	7	1	1
	5.IFN	1	3	5	0.3	2	6	4
HLA-E	1.	2984	-	-	-	2408	1789	1348
	2.	2412	-	-	-	2881	560	1593
	3.	3177	2304	960	1058	2801	1349	1024
	4.	3965	5859	1380	1191	2993	3923	2982
	5.IFN	4021	3903	2122	1347	8706	3846	4141
HLA-F	1.	14	-	-	-	10	13	12
	2.	13	-	-	-	2	2	0.7
	3.	2	5	4	0.8	2	1	3
	4.	2	2	1	0.5	2	0.3	0.2
	5.IFN	0.1	0.8	0.4	0.6	0.3	0.1	0.5
HLA-G	1.	0.1	-	-	-	0.3	0.2	3
	2.	0.2	-	-	-	0	0.1	0.2
	3.	0	0.2	0	0.2	1	0	0.2
	4.	1	3	0.3	0.2	1	0.4	0.2
	5.IFN	0.5	0.3	0	0.3	1	0.2	0.5
B2M	1.	5521	-	-	-	4537	4451	4260
	2.	10399	-	-	-	8944	6058	7060
(Beta-2	3.	1885	3598	2013	3871	2174	2389	3815
-micro	4.	11986	8268	8304	6001	9654	7219	5200
globulin)	5.IFN	4642	4581	3562	4528	3861	17607	14598
TAP1	1.	38	-	-	-	281	157	85
(Peptide.	2.	64	-	-	-	187	120	140
Transp.)	3.	33	75	19	20	132	45	19
	4.	108	538	99	85	430	409	406
	5.IFN	75	188	149	46	3066	1761	1427

Table S2 TLR and MYD88

The five diferent donors are listed as 1-5. Donors 1-4 were treated with 1 ug/ml of *E. coli* LPS and donor 5 with 200 ng/ml of IFN-γ. Donor 1 is a male age 51, donor 2 a male age 43, donor 3 a female age 47, donor 4 a male age 28 and donor 5 a female age 61.

Gene		0	4h	24h	48h	4h+LPS (IFN-γ)	24h+LPS (IFN-γ)	48h+LPS (IFN-γ)
TLR4	1.	24	-	-	-	563	102	138
	2.	44	-	-	-	332	423	78
	3.	25	246	157	193	272	82	74
	4.	19	139	233	220	617	45	24
	5.IFN	18	197	65	202	105	157	166
TLR2	1.	40	-	-	-	306	623	421
	2.	47	-	-	-	539	195	266
	3.	12	86	49	29	59	221	302
	4.	34	165	211	141	211	607	608
	5.IFN	36	299	190	112	93	184	168
TLR8	1.	46	-	-	-	38	209	262
	2.	137	-	-	-	195	150	170
	3.	26	68	65	47	10	145	439
	4.	155	93	92	133	73	195	135
	5.IFN	142	40	96	117	36	377	316
TLR1	1.	6	-	-	-	3	37	36
	2.	27	-	-	-	78	73	69
	3.	11	46	24	9	2	30	84
	4.	10	22	42	13	6	10	16
	5.IFN	3	22	8	15	4	17	19
TLR5	1.	18	-	-	-	26	14	10
	2.	26	-	-	-	8	3	3
	3.	7	18	15	13	7	9	10
	4.	7	4	9	2	13	0.5	0.5
	5.IFN	2	2	2	9	0	3	0.7

Almost no expression of TLR3, TLR6, TLR7, TLR9 and TLR10

MYD88	1.	90	-	-	-	178	81	105
	2.	154	-	-	-	82	113	107
	3.	94	138	78	82	142	80	126
	4.	190	233	161	199	152	99	114
	5.IFN	468	241	163	199	446	592	516

Signaling component for TLRs

Table S3 Complement and Coagulation

The five diferent donors are listed as 1-5. Donors 1-4 were treated with 1 ug/ml of *E. coli* LPS and donor 5 with 200 ng/ml of IFN-γ. Donor 1 is a male age 51, donor 2 a male age 43, donor 3 a female age 47, donor 4 a male age 28 and donor 5 a female age 61.

Gene		0	4h	24h	48h	4h+LPS (IFN-γ)	24h+LPS (IFN-γ)	48h+LPS (IFN-γ)
FCN1	1.	3198	-	-	-	729	15	105
	2.	2681	-	-	-	547	549	18
(Ficolin)	3.	1025	531	679	505	205	3.4	17
	4.	2183	1458	728	614	828	25	65
	5.IFN	3568	1284	2411	1216	1349	879	753
CFP	1.	991	-	-	-	391	307	166
	2.	1017	-	-	-	211	105	140
(Properdin)	3.	542	578	203	97	333	283	146
	4.	784	363	78	13	339	215	134
	5.IFN	1503	1203	383	116	936	259	83
CFB	1.	0.2	-	-	-	429	83	21
	2.	0.1	-	-	-	123	163	15
	3.	2	0.3	1	2	222	17	6.0
	4.	0	0	0	0.5	285	47	24
	5.IFN	0.1	0.4	0.2	0.6	6	64	116
Complement factor B								
C3	1.	2	-	-	-	118	383	555
	2.	1	-	-	-	53	39	89
	3.	2	31	72	46	100	84	135
	4.	0.1	0.7	30	13	29	24	21
	5.IFN	0.2	9	7	36	1	5	17
Complement factor 3								
CFD	1.LPS	249	-	-	-	82	3	8
	2.	196	-	-	-	2.7	4	7
	3.	170	91	74	142	47	2	14
	4.	206	366	47	119	123	8	10
	5.IFN	437	173	200	270	325	81	139
Complement factor D								
F3	1.	0.1	-	-	-	110	4.4	1
	2.	0	-	-	-	506	616	9
	3.	0	2	0	2	717	13	2
	4.	0	0.2	0.7	5	131	49	7
	5.IFN	0	0.6	0.9	0.6	5	0.4	0.4
Coagulation factor 3, Tissue factor								
C1QA	1.	7	-	-	-	0.1	0.6	3
	2.	6	-	-	-	2	7	0.8
	3.	13	7	33	287	2	1	3
	4.	24	5	13	73	4	1	1
	5.IFN	22	9	8	408	10	105	567
Complement factor C1Q- A								

Table S4 Other mixed molecules

The five diferent donors are listed as 1-5. Donors 1-4 were treated with 1 ug/ml of *E. coli* LPS and donor 5 with 200 ng/ml of IFN-γ. Donor 1 is a male age 51, donor 2 a male age 43, donor 3 a female age 47, donor 4 a male age 28 and donor 5 a female age 61. The increase at 4 hrs is marked in black anda t 24 hrs in red.

Gene		0	4h	24h	48h	4h+LPS (IFN-γ)	24h+LPS (IFN-γ)	48h+LPS (IFN-γ)	
SOD2	1.	1113	-	-	-	39601	28919	19446.	36x
	2.	897	-	-	-	16333	3515	8325	
	3.	525	862	194	215	12200	13274	9039	
	4.	1469	4354	777	645	39054	27788	19487	
	5.IFN	566	2288	742	472	3329	2558	2077	
	Superoxide dismutase 2								
ITGB2	1.	1975	-	-	-	443	152	786	
	2.	1423	-	-	-	829	2453	1764	
	3.	685	340	748	802	124	192	1201	
	4.	1504	897	1076	1543	456	148	1037	
	5.IFN	2479	862	3309	1655	1455	1871	2445	
	Integrin beta 2								
ITGB3	1.	3	-	-	-	2	702	287	1x (234x)
	2.	4	-	-	-	154	72	264	
	3.	0.7	4	0.2	0	2	100	10	
	4.	1	5	0.7	0.1	2	319	430	
	5.IFN	2	1	0	0.3	1	0	0	
	Integrin beta 3								
ITGB8	1.	0	-	-	-	206	621	432	
	2.	0	-	-	-	352	40	141	
	3.	0	0.7	0.4	0.6	202	317	91	
	4.	0	0.5	0.7	0	107	402	164	
	5.IFN	0	0.6	0.4	0.5	0	0.1	0	
	Integrin beta 8								
CRIM1	1.	0.8	-	-	-	441	513	336	551x
	2.	1	-	-	-	815	981	1111	
	3.	0.9	7	12	10	269	67	22	
	4.	3	7	103	31	268	668	753	
	5.IFN	2	25	29	17	18	10	4	
	Cystein rich Transmembrane BMP regulator 1								
NFKBIA	1.	1347	-	-	-	4063	2666	1338	
	2.	591	-	-	-	3705	3480	1439	
	3.	2370	897	308	268	6131	2681	1402	
	4.	900	899	289	160	2720	2489	1642	
	5.IFN	2415	1238	605	391	1835	357	114	
	NFkB component								
PLA2G7	1.	15	-	-	-	284	425	602	
	2.	3	-	-	-	800	545	633	
	3.	17	93	282	527	303	545	515	
	4.	26	71	598	390	185	460	283	
	5.IFN	10	149	103	436	43	178	124	
	Phospolipase A2								

GCH1	1.	8	-	-	-	830	184	52	
	2.	14	-	-	-	543	506	107	
	3.	8	21	2	2	363	244	113	
	4.	10	22	4	3	610	114	30	
	5.IFN	15	43	1	4	257	278	258	
	GTP cyclohydrolase 1								
OLR1	1.	2	-	-	-	1415	63	105	
	2.	1	-	-	-	194	620	325	
	3.	3	964	168	41	474	124	163	
	4.	0.6	878	755	303	1117	49	119	
	5.IFN	0.5	864	209	88	22	18	11	
	Oxidized low density lipoprotein receptor								
IDO1	1.	0.1	-	-	-	101	382	279	1010x (3820x)
	2.	0	-	-	-	850	270	547	
	3.	0	0.2	0.4	0.3	58	272	143	
	4.	0.8	1.0	0.2	0.1	161	1820	1595	
	5.IFN	0	0.1	1.9	0.2	650	4901	2867	
	Indole amine 2-3 dioxidase 1								
SLAMF7	1.	2	-	-	-	2987	2136	1301	
	2.	6	-	-	-	1385	441	926	
	3.	2	67	16	33	910	433	516	
	4.	31	247	212	120	2914	2243	1033	
	5.IFN	9	93	24	43	301	1203	958	
	SLAM family 7								
SGPP2	1.	0.1	-	-	-	701	420	236	
	2.	0.1	-	-	-	306	108	201	
	3.	0	5	2	4	747	99	21	
	4.	0	8	4.0	1	514	631	386	
	5.IFN	9	93	24	43	301	1203	958	
	Shingosine-1- phosphate Phosphatase 2								
SRGN	1.	3855	-	-	-	6736	1934	2185	
	2.	3641	-	-	-	3899	2312	3170	
(Serglycin)	3.	1310	1617	287	337	2431	1332	1266	
	4.	5214	5595	1432	1123	10261	4215	3187	
	5.IFN	4756	1080	961	710	2326	1146	1047	
ZC3HAV1	1.	41	-	-	-	968	111	54	
	2.	70	-	-	-	910	1158	65	
	3.	22	62	37	43	583	29	30	
	4.	47	198	92	119	1362	234	159	
	5.IFN	89	120	177	89	876	179	143	
	Antiviral Zinc finger								
ZC3H12C	1.	2	-	-	-	274	147	112	
	2.	4	-	-	-	215	228	39	
	3.	3	29	24	37	171	38	27	
	4.	3	52	32	59	312	248	118	
	5.IFN	8	86	30	60	44	69	36	
	Zinc finger								
MX2	1.	14	-	-	-	338	25	13	
	2.	10	-	-	-	115	200	5	
	3.	20	11	4	7	292	8	5	
	4.	4	2	5	1	126	8	1	
	5.IFN	2	1	0	1	2	4	4	
	Myxo-virus resistance								

**MIR155HG**	**1.**	**0.1**	**-**	**-**	**-**	**182**	**81**	**9**
	**2.**	**0.2**	**-**	**-**	**-**	**387**	**382**	**74**
	**3.**	**0**	**1**	**0**	**2**	**287**	**7.6**	**0.5**
	**4.**	**0.1**	**1**	**2**	**0.7**	**256**	**174**	**10**
	**5.IFN**	**0.3**	**3**	**0.2**	**2**	**4**	**1**	**0.1**
	**Non coding RNA**							
**RNASE1**	**1.**	**0.6**	**-**	**-**	**-**	**0.7**	**1**	**3**
	**2.**	**0.2**	**-**	**-**	**-**	**5**	**24**	**22**
	**3.**	**0.9**	**5**	**62**	**176**	**0**	**0**	**0.6**
	**4.**	**0.4**	**5**	**77**	**248**	**0**	**2**	**38**
	**5.IFN**	**3**	**25**	**784**	**1894**	**2**	**3**	**11**
	**Ribonuclease 1 pancreatic**							

Table S5 Proteases and protease inhibitors

The five diferent donors are listed as 1-5. Donors 1-4 were treated with 1 ug/ml of *E. coli* LPS and donor 5 with 200 ng/ml of IFN-γ. Donor 1 is a male age 51, donor 2 a male age 43, donor 3 a female age 47, donor 4 a male age 28 and donor 5 a female age 61.

Gene		0	4h	24h	48h	4h+LPS (IFN-γ)	24h+LPS (IFN-γ)	48h+LPS (IFN-γ)
MMP14	1.	6	-	-	-	759	4051	3268
	2.	6	-	-	-	2002	950	1886
	3.	3	28	103	96	659	2442	1333
	4.	7	18	181	193	286	3012	2402
	5.IFN	5	22	133	186	4	35	39
	Matrix metallo peptidase 14							
CTSS	1.	5290	-	-	-	1587	930	1194
	2.	5923	-	-	-	343	642	452
	3.	1024	861	818	1443	366	198	418
	4.	2853	559	1283	1392	1724	264	153
	5.IFN	1278	455	234	2056	188	965	1428
	Cathepsin S involved in antigen presentation to MHC Class II							
Cystatin C	1.	3704	-	-	-	985	114	142
	2.	4034	-	-	-	1197	954	207
(CST3)	3.	2446	1905	2258	3887	551	118	283
	4.	4906	2961	1692	2398	1316	167	158
	5.IFN	7478	2667	3147	4974	3650	1952	2850
SerpinB2	1.	38	-	-	-	7326	17175	3847
	2.	10	-	-	-	7380	350	1880
	3.	1	6	3	3.2	928	1459	85
	4.	5	8	6	0.7	1305	3098	1051
	5.IFN	2	21	0.2	5	16	0	0
SerpinB9	1.	111	-	-	-	2491	948	276
	2.	59	-	-	-	1326	1318	707
	3.	28	76	7	7	535	608	353
	4.	16	20	10	1	712	35	8
	5.IFN	4	23	0.2	4	10	14	6
PI3	1.	2	-	-	-	582	5620	2384
	2.	0	-	-	-	43	7	44
	3.	2	33	2.7	1.0	189	326	221
	4.	0	8	0	0	93	2382	1371
	5.IFN	0.4	6	0	0	3	2	0.7
	Peptidase inhibitor 3 skin derived							

Table S6 CD molecules

The five diferent donors are listed as 1-5. Donors 1-4 were treated with 1 ug/ml of *E. coli* LPS and donor 5 with 200 ng/ml of IFN-γ. Donor 1 is a male age 51, donor 2 a male age 43, donor 3 a female age 47, donor 4 a male age 28 and donor 5 a female age 61.

Gene		0	4h	24h	48h	4h+LPS (IFN-γ)	24h+LPS (IFN-γ)	48h+LPS (IFN-γ)
CD14	1.	1697	-	-	-	574	7300	7946
	2.	1633	-	-	-	2360	1119	2477
	3.	991	904	2245	1780	230	6021	7424
	4.	1525	1010	2143	1661	256	2243	8437
	5.IFN	3869	1596	4229	2458	989	236	531
CD4	1.	594	-	-	-	88	8	14
	2.	578	-	-	-	4	24	6
	3.	81	105	226	274	12	2	17
	4.	564	237	174	614	105	14	10
	5.IFN	1422	320	895	1278	308	442	802
CD83	1.	18	-	-	-	565	231	113
	2.	18	-	-	-	575	350	538
	3.	44	88	53	103	700	72	42
	4.	39	336	186	314	1054	980	791
	5.IFN	161	362	120	187	809	235	79
CD80	1.	2	-	-	-	318	146	56
	2.	0.4	-	-	-	252	76	101
	3.	2	8	3	5	632	161	70
	4.	1	6	2	0	130	165	53
	5.IFN	0.8	14	0.4	2	51	51	23
CD86	1.	236	-	-	-	78	4	33
	2.	207	-	-	-	20	131	85
	3.	34	72	40	48	15	2	19
	4.	152	145	67	124	101	5	15
	5.IFN	108	47	28	82	50	97	114
CD274	1.	0	-	-	-	139	551	214
	2.	0.5	-	-	-	271	118	150
	3.	0.2	6	3	3	146	202	166
	4.	5	8	8	5	247	687	211
	5.IFN	0.2	15	5	8	423	373	253
CD34	1.	0.1	-	-	-	0	0	0
	2.	0	-	-	-	0	0	0.2
	3.	0	0	0	0	0	0	0.2
	4.	0	0	0.1	0.1	0.1	0	0
	5.IFN	0	0	0	0	0	0	0
CD19	1.	0	-	-	-	0.1	0.2	2.8
	2.	0	-	-	-	0	0.2	0.1
	3.	0	0	0	0	0	0	0
	4.	0	0	0	0	0	0.1	0
	5.IFN	0	0	0	0	0	0	0

Table S7 Immunoglobulin Fc-receptors

The five diferent donors are listed as 1-5. Donors 1-4 were treated with 1 ug/ml of *E. coli* LPS and donor 5 with 200 ng/ml of IFN-γ. Donor 1 is a male age 51, donor 2 a male age 43, donor 3 a female age 47, donor 4 a male age 28 and donor 5 a female age 61.

Gene		0	4h	24h	48h	4h+LPS (IFN-γ)	24h+LPS (IFN-γ)	48h+LPS (IFN-γ)
FCGR2A	1.	580	-	-	-	343	797	580
	2.	404	-	-	-	707	422	716
	3.	55	111	53	46	107	258	398
	4.	756	3676	351	398	396	899	3456
	5.IFN	1243	350	3450	305	469	545	493
FCGR3A	1.	131	-	-	-	6	22	36
	2.	93	-	-	-	6	15	5
	3.	27	123	74	20	6	3	62
	4.	150	611	403	219	16	8	51
	5.IFN	297	207	340	10	207	249	130
FCGR3B	1.	22	-	-	-	4	1	3
	2.	1	-	-	-	0	0	0
	3.	23	15	0.4	0.6	3	0.5	0
	4.	3	5	0.2	0	0.2	0.4	0
	5.IFN	6	0.3	0	0.2	2	1	0.5
FCERIA	1.	7	-	-	-	0.3	0.2	0
	2.	41	-	-	-	0	0	0
	3.	5	1	17	32	0	0	0
	4.	24	10	20	20	1	0.1	0
	5.IFN	34	6	49	62	4	0.1	0
FCERIG	1.	1173	-	-	-	1048	3435	3033
	2.	1426	-	-	-	4407	3504	3764
	3.	567	993	845	1399	549	3675	4134
	4.	2096	2724	2072	2401	1721	4966	4665
	5.IFN	2125	1526	1685	2537	1360	3120	2888
FCER2	1.	2	-	-	-	2	2	3
(CD23)	2.	1	-	-	-	2	1	1
	3.	4	8	3	3	7	2	3
	4.	0.3	3	1	1	1	2	3
	5.IFN	3	5	0.2	1	8	0.3	0.7
FCGR1A	1.	51	-	-	-	10	25	70
	2.	39	-	-	-	8	3	7
	3.	13	13	7	4	1	47	125
	4.	133	89	50	62	56	8	6
	5.IFN	60	23	19	16	397	705	764
FCAR	1.	92	-	-	-	344	426	293
(CD89)	2.	33	-	-	-	285	214	287
	3.	27	51	11	4	148	276	225
	4.	18	34	44	1	213	64	81
	5.IFN	11	46	3	1	2	3	0.4

Table S8. Cytokines and Chemokines

The five diferent donors are listed as 1-5. Donors 1-4 were treated with 1 ug/ml of *E. coli* LPS and donor 5 with 200 ng/ml of IFN-γ. Donor 1 is a male age 51, donor 2 a male age 43, donor 3 a female age 47, donor 4 a male age 28 and donor 5 a female age 61. The increase at 4 hrs is marked in black anda t 24 hrs in red.

Gene		0	4h	24h	48h	4h+LPS (IFN-γ)	24h+LPS (IFN-γ)	48h+LPS (IFN-γ)	Increase
IL-1a	1.	0.2	-	-	-	2134	1049	421	10670x
	2.	0.1	-	-	-	1735	1920	455	
	3.	0	5	0.2	0	1117	388	178	
	4.	0	6	4	0.9	2745	1856	744	
	5.IFN	0.1	26	0.2	0.6	1	0	0.7	
IL-1b	1.	11	-	-	-	31674	23094	12926	2879x
	2.	16	-	-	-	32115	31994	18578	
	3.	9	62	15	5	10085	12696	6181	
	4.	12	72	48	8	43189	31663	14788	
	5.IFN	48	234	2	3	19	16	4	
IL-6	1.	0.1	-	-	-	5850	1717	1071	58500x
	2.	0.1	-	-	-	10270	13580	775	
	3.	0.2	2	2	0.2	5712	1775	443	
	4.	0	1	1	0	8386	3175	1126	
	5.IFN	0.3	35	0	0.1	7	0.1	0	
TNF-a	1.	131	-	-	-	2047	310	94	16x
	2.	91	-	-	-	3391	4672	366	
(TNF)	3.	72	67	52	26	1173	61	52	
	4.	86	61	71	45	2955	248	74	
	5.IFN	295	228	81	44	1114	433	202	
IL-10	1.	4	-	-	-	42	628	141	10x (157x)
	2.	0.7	-	-	-	211	12	53	
	3.	1	6	9	19	36	230	34	
	4.	0.8	4	31	26	22	457	100	
	5.IFN	0.9	4	11	28	0.3	4	7	
IL-19	1.	0.1	-	-	-	12	591	70	120x (5910x)
	2.	0	-	-	-	156	5.3	30	
	3.	0	0	0	0	25	22	3	
	4.	0	0	0.3	0	3	768	76	
	5.IFN	0	0	0	0	0	0	0	
IL-20	1.	0	-	-	-	2	12	5	20x (120x)
	2.	0	-	-	-	3	0	0.2	
	3.	0	0.2	0	0	3	0.4	0.3	
	4.	0	0	0.3	0	6	58	3.3	
	5.IFN	0	0	0.4	0	0	0	0	
IL-36G	1.	0	-	-	-	342	765	154	3420x (7650x)
	2.	0	-	-	-	265	2	24	
	3.	0.2	0	0	0	387	96	32	
	4.	0	0.5	0.2	0	246	1106	101	
	5.IFN	0	0	0	0	0.2	0	0	

**IL-12B**	**1.**	**0**	**33**	**16**	**11**	**27**	**15**	**5**
	**2.**	**0.1**	**31**	**34**	**63**	**87**	**12**	**9**
	**3.**	**0**	**0**	**0**	**0**	**14**	**3**	**0.3** No increase in IL12A
	**4.**	**0**	**0**	**0**	**0**	**30**	**234**	**12** No increase in IL12A
	**5.IFN**	**0**	**0**	**0**	**0**	**0.3**	**0**	**0**
**CSF3**	**1.**	**0**	**-**	**-**	**-**	**221**	**587**	**241**
	**2.**	**0**	**-**	**-**	**-**	**105**	**143**	**6**
(G-CSF)	**3.**	**0**	**0**	**0**	**0**	**316**	**201**	**31**
	**4.**	**0**	**0**	**0**	**0**	**244**	**776**	**356**
	**5.IFN**	**0**	**0.2**	**0**	**0**	**0**	**0**	**0**
**TGFBI**	**1.**	**1297**	**-**	**-**	**-**	**206**	**18**	**211** Down regulated (72x)
**(Induced)**	**2.**	**778**	**-**	**-**	**-**	**171**	**187**	**202**
	**3.**	**287**	**2096**	**2217**	**1194**	**98**	**92**	**414**
	**4.**	**739**	**1461**	**3597**	**2959**	**200**	**21**	**95**
	**5.IFN**	**1129**	**1180**	**3672**	**992**	**3722**	**2204**	**2147**
**IL1RN**	**1.**	**55**	**-**	**-**	**-**	**1482**	**1421**	**561**
	**2.**	**27**	**-**	**-**	**-**	**1406**	**1171**	**1237**
	**3.**	**17**	**16**	**5**	**38**	**273**	**186**	**156**
	**4.**	**38**	**45**	**53**	**128**	**1196**	**787**	**251**
	**5.IFN**	**45**	**34**	**9**	**38**	**27**	**184**	**84**

IL-1 receptor antagonist

**TNIP3**	**1.**	**0.2**	**-**	**-**	**-**	**765**	**1882**	**760**
	**2.**	**0**	**-**	**-**	**-**	**525**	**182**	**307**
	**3.**	**0**	**1**	**0.8**	**0**	**188**	**573**	**529**
	**4.**	**0**	**3**	**3**	**0.2**	**498**	**714**	**366**
	**5.IFN**	**0.1**	**2**	**0.4**	**0.1**	**1**	**0.1**	**0.3**

TNFAIP3 interacting protein, Inhibits NFkB activation by TNF-a, IL-1 and TLR-4

No expression of IL-2, IL-3, IL-4, IL-5, IL-9, IL-21, IL-22, IL-25, IL-36, IL-28, IL-31, IL-33, IL-34, IL-36A, IL-36B and IL-37. In a few a very minor expression at 48 h +LPS.

**IL-8**	**1.**	**1202**	**-**	**-**	**-**	**43405**	**38170**	**44709**	**36x**
	**2.**	**174**	**-**	**-**	**-**	**55515**	**52850**	**27652**	
	**3.**	**1034**	**727**	**149**	**88**	**36945**	**21832**	**10355**	
	**4.**	**176**	**181**	**741**	**76**	**46829**	**24100**	**12508**	
	**5.IFN**	**129**	**587**	**19**	**48**	**52**	**9**	**1**	
**CCL1**	**1.**	**0**	**-**	**-**	**-**	**3**	**6**	**7**	**30x (60x)**
	**2.**	**0**	**-**	**-**	**-**	**1787**	**1504**	**1974**	**17870 (15040x)**
	**3.**	**0**	**0**	**0.6**	**0**	**2**	**0.5**	**0.6**	
	**4.**	**0**	**0.5**	**0.8**	**0**	**9**	**649**	**587**	
	**5.IFN**	**0**	**0.3**	**0.6**	**0**	**0.9**	**0.3**	**0**	
**CCL2**	**1.**	**2**	**-**	**-**	**-**	**1143**	**22852**	**24177**	**571x (11426x)**
	**2.**	**2**	**-**	**-**	**-**	**11791**	**4575**	**10308**	
	**3.**	**6**	**155**	**98**	**136**	**6235**	**13541**	**12482**	
	**4.**	**2**	**278**	**1313**	**403**	**1336**	**19071**	**18286**	
	**5.IFN**	**16**	**408**	**213**	**64**	**1707**	**524**	**117**	
**CCL3**	**1.**	**9**	**-**	**-**	**-**	**8722**	**9090**	**3458**	**969x**
	**2.**	**14**	**-**	**-**	**-**	**12384**	**15015**	**2645**	
	**3.**	**11**	**37**	**24**	**24**	**9660**	**2372**	**556**	
	**4.**	**7**	**13**	**33**	**16**	**11498**	**5280**	**1000**	
	**5.IFN**	**18**	**64**	**2**	**17**	**12**	**6**	**3**	

CCL3L3	1.	2	-	-	-	774	1288	408	387x
	2.	0.6	-	-	-	1016	1159	161	
	3.	2	8	3	2	1868	332	57	
	4.	5	30	6	3	2877	3669	2549	
	5.IFN	18	20	6	2	12	1	21	
CCL4	1.	3	-	-	-	16893	6716	2349	5631x
	2.	3	-	-	-	22220	25169	2583	
	3.	1	27	12	20	10084	1394	442	
	4.	4	58	33	29	20638	9367	3106	
	5.IFN	8	439	54	42	203	34	14	
CCL5	1.	8	-	-	-	450	60	39	56x
	2.	7	-	-	-	416	461	52	
	3.	4	3	3	4	140	8	8	
	4.	15	32	49	31	541	94	87	
	5.IFN	13	13	17	54	6	12	5.2	
CCL7	1.	0.3	-	-	-	288	1108	1265	960x (3693x)
	2.	0.4	-	-	-	408	319	630	
	3.	0.2	5	0	0	126	86	140	
	4.	0.3	88	15	1	272	881	975	
	5.IFN	1	43	0.6	0.1	139	2	0	
CCL8	1.	0	-	-	-	39	55	40	390x (550x)
	2.	0	-	-	-	20	1	4	
	3.	0	0.5	0	0.2	12	6	4	
	4.	0	0	0.2	0	3	4	1	
	5.IFN	0	2	6	42	0.2	0	0	
CCL13	1.	0	-	-	-	0.3	12	12	3x (120x)
	2.	0	-	-	-	20	13	22	
	3.	0	0	0	0	0	0.9	0.5	
	4.	0	0	0	0.1	0.4	24	75	
	5.IFN	0	0	0.6	0	5	3	0.7	
CCL15	1.	0	-	-	-	51	15	1	510x
	2.	0.1	-	-	-	72	65	6	
	3.	0	0	0	0	11	0.4	0	
	4.	0	0	0	0	0	48	27	
	5.IFN	0	1	0	0	1	0	0	
CCL18	1.	0	-	-	-	76	355	87	760x (3550x)
	2.	0	-	-	-	18	14	13	
	3.	0	0.5	0	0.5	18	25	11	
	4.	0	27	1	4	68	180	55	
	5.IFN	0	4	4	8	26	13	4	
CCL19	1.	0	-	-	-	3	103	43	30x (1030x)
	2.	0.1	-	-	-	52	29	24	
	3.	0.3	0	0	0	2	39	17	
	4.	0	0	0.2	0	2	67	39	
	5.IFN	0.1	0.4	0	0	0	1	0.6	
CCL20	1.	0.4	-	-	-	2662	269	194	6655x
	2.	0.2	-	-	-	3680	4151	208	
	3.	0	2	0.2	0	3515	256	63	
	4.	0	0	1	0.5	5585	539	49	
	5.IFN	0.1	5	0	0	0.6	0	0.4	

CCL21	1.	0	-	-	-	3	0.5	17	30x (5x)
	2.	0	-	-	-	0	0	0.1	
	3.	0	0	0.2	0	0	0.2	0.3	
	4.	0	0	0.2	0	0.1	0	0	
	5.IFN	0	0.3	0	0	0	0.1	0	
CCL22	1.	0	-	-	-	31	209	112	310x (2090x)
	2.	0.1	-	-	-	5979	8926	11219	
	3.	0.2	0.7	2	6	13	25	12	
	4.	0	11	9	5	109	1113	571	
	5.IFN	0.6	15	18	24	4	4	0.9	
CCL23	1.	0.2	-	-	-	37	32	6	185x
	2.	0.4	-	-	-	15	4	2	
	3.	0	0	0.8	0	24	21	7	
	4.	0.1	1	0.2	0.9	31	32	12	
	5.IFN	0.1	1	1	0.5	2	0.7	0	
CCL24	1.	0.8	-	-	-	8	179	283	10x (224x)
	2.	0.6	-	-	-	10974	15748	12733	18290x
	3.	0	3	22	9	16	17	50	
	4.	0.1	4	157	5	8	3557	4104	
	5.IFN	1	36	300	23	3	4	0	
CCL27	1.	0.1	-	-	-	0	0	1.2	-
	2.	0	-	-	-	0	0	0	
	3.	0	0	0.2	0	0	0	0	
	4.	0	0	0	0	0	0	0	
	5.IFN	0	0	0	0	0	0	0	

No or very low expression of CCL11, CCL14, CCL16, CCL17, CCL25, CCL26, CCL27, CCL28.

CXCL1	1.	4	-	-	-	1249	6646	6196	312x (1661x)
	2.	2	-	-	-	3525	523	1089	
	3.	4	23	7	3	2572	8503	7648	
	4.	0.4	0.7	3	0.1	190	228	227	
	5.IFN	0	3	0.2	0.3	3	0	0	
CXCL2	1.	12	-	-	-	1763	1615	702	147x
	2.	3	-	-	-	1006	95	222	
	3.	6	35	76	108	5819	2331	970	
	4	3	4	27	4	1367	3001	58	
	5.IFN	0.7	20	2	21	0.8	0.8	0.7	
CXCL3	1.	0.3	-	-	-	749	2306	1863	2497x (7687x)
	2.	0.2	-	-	-	2907	464	1640	
	3.	0.3	17	20	25	1833	3836	2825	
	4.	0.7	5	19	7	1771	3421	1722	
	5.IFN	1	69	8	27	7	0.1	0	
CXCL5	1.	0	-	-	-	32	6651	13897	320x (66510x)
	2.	0.2	-	-	-	10768	7542	12612	
	3.	0.5	2	6	0.3	45	13957	15443	
	4.	0.4	0.7	5	0.6	16	3097	4329	
	5.IFN	0	4	0.2	0.4	0.2	0	0	

**CXCL6 1.**	**0.1**	**-**	**-**	**-**	**4**	**70**	**88**	**40x (700x)**
**2.**	**0**	**-**	**-**	**-**	**473**	**37**	**105**	
**3.**	**0.2**	**3**	**0.8**	**0**	**16**	**1075**	**312**	
**4.**	**0.6**	**0.2**	**2**	**0.7**	**2**	**113**	**118**	
**5.IFN**	**0.2**	**2**	**0.4**	**0.1**	**2**	**0**	**0**	
**CXCL10 1.**	**0.2**	**-**	**-**	**-**	**192**	**0.6**	**0**	**960x**
**2.**	**0.8**	**-**	**-**	**-**	**106**	**283**	**0.1**	
**3.**	**1**	**5**	**0**	**0.3**	**157**	**0**	**0**	
**4.**	**4**	**1**	**0.2**	**0**	**151**	**0.1**	**0**	
**5.IFN**	**2**	**21**	**0.4**	**0.4**	**1554**	**1619**	**855**	
**CXCL11 1.**	**0**	**-**	**-**	**-**	**11**	**0**	**0**	**110x**
**2.**	**0.6**	**-**	**-**	**-**	**0.1**	**0**	**0**	
**3.**	**0.7**	**0.3**	**0**	**0**	**30**	**0.4**	**0**	
**4.**	**2**	**2**	**0**	**0**	**40**	**2**	**0**	
**5.IFN**	**0.2**	**4**	**0.6**	**0**	**1494**	**363**	**83**	
**CXCL16 1.**	**307**	**-**	**-**	**-**	**196**	**199**	**509**	-
**2.**	**134**	**-**	**-**	**-**	**1033**	**1124**	**1213**	
**3.**	**55**	**231**	**67**	**43**	**57**	**125**	**362**	
**4.**	**432**	**1063**	**310**	**276**	**332**	**495**	**1089**	
**5.IFN**	**294**	**972**	**441**	**164**	**1838**	**669**	**349**	
**IRAK2 1.**	**5**	**-**	**-**	**-**	**302**	**222**	**89**	
**2.**	**3**	**-**	**-**	**-**	**82**	**25**	**51**	
**3.**	**4**	**29**	**4**	**3**	**557**	**139**	**50**	
**4.**	**34**	**14**	**8**	**2**	**242**	**129**	**40**	
**5.IFN**	**5**	**46**	**2**	**4**	**10**	**3**	**0.3**	
**INHBA 1.**	**1**	**-**	**-**	**-**	**80**	**761**	**304**	-
**2.**	**0**	**-**	**-**	**-**	**1745**	**425**	**906**	
**3.**	**0.2**	**0.7**	**0.2**	**0.6**	**254**	**100**	**20**	
**4.**	**0**	**0**	**2**	**0**	**165**	**1410**	**708**	
**5.IFN**	**0.2**	**0.7**	**0.2**	**0**	**b5**	**6**	**3**	

Inhibin Beta A

No expression (or increase) of CXCL6, CXCL9, CXCL12, CXCL13, CXCL14, CXCL17.

Table S9 Cytokine and other receptors

The five diferent donors are listed as 1-5. Donors 1-4 were treated with 1 ug/ml of *E. coli* LPS and donor 5 with 200 ng/ml of IFN-γ. Donor 1 is a male age 51, donor 2 a male age 43, donor 3 a female age 47, donor 4 a male age 28 and donor 5 a female age 61.

Gene		0	4h	24h	48h	4h+LPS (IFN-γ)	24h+LPS (IFN-γ)	48h+LPS (IFN-γ)
IL15RA	1.	14	-	-	-	92	33	13
	2.	29	-	-	-	52	13	21
	3.	25	15	2	6	289	52	12
	4.	28	4	2	2	101	70	23
	5.IFN	15	6	0.2	2	80	60	36
IL7R	1.	0.7	-	-	-	490	1561	792
	2.	2	-	-	-	598	650	605
	3.	2	4	5	8	141	383	210
	4.	4	14	28	35	422	963	329
	5.IFN	3	15	12	58	4	7	5
IL2RA	1.	0.1	-	-	-	52	342	71
	2.	0	-	-	-	383	66	135
	3.	0	0.2	0	0	67	333	38
	4.	0	3	1	0.6	70	673	331
	5.IFN	0.3	0.6	1	0.9	76	2	3
IL10RA	1.	609	-	-	-	264	153	243
	2.	443	-	-	-	295	330	274
	3.	357	531	406	313	420	349	477
	4.	422	222	198	115	281	158	151
	5.IFN	237	237	126	171	175	180	115
IL17RA	1.	225	-	-	-	53	353	340
	2.	111	-	-	-	205	339	334
	3.	256	211	207	99	62	263	247
	4.	158	147	302	189	59	178	224
	5.IFN	383	281	272	230	226	314	357
CCR7	1.	0.6	-	-	-	223	208	125
	2.	0.7	-	-	-	425	404	798
	3.	0	4	0.6	3	53	27	27
	4.	0.8	14	6	5	391	780	726
	5.IFN	1	13	10	19	6	4	2
ADORA2A	1.	14	-	-	-	666	566	413
	2.	4	-	-	-	404	403	270
	3.	9	14	2	0.5	476	287	200
	4.	4	8	3	0.5	348	268	171
	5.IFN	5	29	0.6	0.9	6	0.9	1

Adenosine receptor A2

Table S10 Genes upregulated by IFN-γ

Gene		0	4h	24h	48h	4h+IFN-γ	24h+IFN-γ	48h+IFN-γ
**GBP1**	**5.IFN**	**6**	**18**	**3**	**3**	**670**	**1328**	**813**
	(Guanylate binding IFN induced)							
**GBP5**	**5.IFN**	**34**	**19**	**4**	**9**	**6018**	**3915**	**3153**
	(Guanylate binding IFN induced)							
**C1QB**	**5.IFN**	**13**	**4**	**73**	**266**	**85**	**873**	**2709**
**IDO1**	**5.IFN**	**0**	**0.1**	**2**	**0.2**	**650**	**4901**	**2867**
	Indole amine 2-3 dioxidase 1							
**WARS**	**5.IFN**	**519**	**310**	**45**	**85**	**3976**	**2746**	**2522**
	(Aralkylam. N-acetyl transferase)							
**RSAD2**	**5.IFN**	**4**	**35**	**4**	**1**	**654**	**467**	**412**
	(Viperin Virus inh.)							
**CCL7**	**5.IFN**	**1**	**43**	**0.6**	**0.1**	**139**	**2**	**0**
**CXCL9**	**5.IFN**	**3**	**1**	**1**	**1**	**632**	**6084**	**4671**
**CXCL10**	**5.IFN**	**2**	**21**	**0.4**	**0.4**	**1554**	**1619**	**855**
**CXCL11**	**5.IFN**	**0.2**	**4**	**0.6**	**0**	**1494**	**363**	**83**
**SLAMF7**	**5.IFN**	**9**	**93**	**24**	**43**	**301**	**1203**	**958**
	(SLAM family member 7 Plasma cell marker CD319)							
**SLAMF8**	**5.IFN**	**2**	**18**	**199**	**220**	**607**	**1076**	**1105**
	(CD2 family member involved in lymphocyte activation)							
**ANKRD22**	**5.IFN**	**2**	**1**	**0.2**	**0.5**	**499**	**796**	**566**
	(Ankyrin repeat domain 22)							
**APOL1**	**5.IFN**	**8**	**8**	**6**	**8**	**184**	**158**	**214**
	(Apolipoprotein like 1)							
**APOL4**	**5.IFN**	**0.2**	**0.6**	**0**	**0.7**	**254**	**319**	**315**
	(Apolipoprotein like 4)							
**SERPING1**	**5.IFN**	**15**	**3**	**5**	**14**	**215**	**1539**	**2732**
	(Protease Inh.)							
**VAMP5**	**5.IFN**	**16**	**2**	**4**	**4**	**209**	**264**	**286**
	(Vesicle-associated membrane protein 5)							
**HAPLN3**	**5.IFN**	**0.9**	**5**	**0.8**	**1**	**136**	**253**	**249**
	(hyaluronan and proteoglycan link protein 3)							
**IL27**	**5.IFN**	**12**	**3**	**0.4**	**0.9**	**129**	**74**	**61**
**ETV7**	**5.IFN**	**0.1**	**0.5**	**0.2**	**0**	**116**	**72**	**60**
	(Transcription factor ETV7)							
**P2RY14**	**5.IFN**	**0.5**	**0**	**0**	**0**	**46**	**137**	**158**
	(P2Y purino receptor 14)							

Table S11 Additional Cytokines and Chemokines

The five diferent donors are listed as 1-5. Donors 1-4 were treated with 1 ug/ml of *E. coli* LPS and donor 5 with 200 ng/ml of IFN-γ. Donor 1 is a male age 51, donor 2 a male age 43, donor 3 a female age 47, donor 4 a male age 28 and donor 5 a female age 61. The increase at 4 hrs is marked in black and at 24 hrs in red.

Gene		0	4h	24h	48h	4h+LPS (IFN-γ)	24h+LPS (IFN-γ)	48h+LPS (IFN-γ)	Increase
IL-2	1.	0	-	-	-	0	0	0.7	
	2.	0	-	-	-	0	0	0	
	3.	0	0	0	0	0	0	0	
	4.	0	0	0	0	0	0	0	
	5.IFN	0	0	0	0	0	0	0	
IL-3	1.	0	-	-	-	0	0	6	
	2.	0	-	-	-	0	0	0	
	3.	0	0	0	0	0	0	0.2	
	4.	0	0	0	0	0	0	0	
	5.IFN	0	0	0	0	0	0	0	
IL-4	1.	0	-	-	-	0	0	0	
	2.	0	-	-	-	0	0	0	
	3.	0	0	0	0	0	0	0	
	4.	0	0	0	0	0	0.3	0	
	5.IFN	0	0	0	0	0	0	0	
IL-5	1.	0	-	-	-	0.1	0	8	
	2.	0	-	-	-	0	0	0	
	3.	0	0	0	0	0	0	0	
	4.	0	0	0	0	0	0	0	
	5.IFN	0	0	0	0.1	0	0	0	
IL-9	1.	0	-	-	-	0.3	0	0	
	2.	0	-	-	-	0.1	0	0	
	3.	0	0	0	0.8	0	0	0	
	4.	0	0	0	0	0.1	0	0	
	5.IFN	0	0	0	0	0	0	0	
IL-21	1.	0	-	-	-	0	0	0	
	2.	0	-	-	-	0	0	0	
	3.	0	0	0	0	0	0	0	
	4.	0	0	0	0.1	0	0	0	
	5.IFN	0	0	0	0	0	0	0	
IL-22	1.	0	-	-	-	0	0	0.2	
	2.	0	-	-	-	0	0	0	
	3.	0.5	0	0	0	0	0	0	
	4.	0	0	0	0	0	0	0	
	5.IFN	0	0	0	0	0.2	0	0	
IL-25	1.	0	-	-	-	0.1	0	9	
	2.	0	-	-	-	0	0	0	
	3.	0	0	0	0	0	0	0	
	4.	0	0	0	0	0	0	0	
	5.IFN	0	0	0	0	0	0	0	

**IL-28**	**1.**	**0**	**-**	**-**	**-**	**0**	**0**	**1**
	**2.**	**0.2**	**-**	**-**	**-**	**0.1**	**0**	**0**
	**3.**	**0**	**0**	**0**	**0**	**0**	**0**	**0**
	**4.**	**0.8**	**0**	**0**	**0**	**0**	**0**	**0**
	**5.IFN**	**0**	**0**	**0**	**0**	**0**	**0**	**0**
**IL-31**	**1.**	**0**	**-**	**-**	**-**	**0**	**0**	**0**
	**2.**	**0**	**-**	**-**	**-**	**0**	**0**	**0**
	**3.**	**0**	**0**	**0**	**0**	**0**	**0**	**0**
	**4.**	**0**	**0**	**0**	**0**	**0**	**0**	**0**
	**5.IFN**	**0**	**0**	**0**	**0**	**0**	**0**	**0**
**IL-33**	**1.**	**0**	**-**	**-**	**-**	**0**	**0**	**0**
	**2.**	**0**	**-**	**-**	**-**	**0**	**0**	**0.1**
	**3.**	**0**	**0**	**0**	**0**	**0**	**0**	**0**
	**4.**	**0**	**0**	**0**	**0**	**0**	**0**	**0**
	**5.IFN**	**0**	**0**	**0**	**0**	**0**	**0**	**0**
**IL-34**	**1.**	**0**	**-**	**-**	**-**	**0.1**	**0**	**2**
	**2.**	**0**	**-**	**-**	**-**	**0**	**0**	**0**
	**3.**	**0**	**0**	**0**	**0**	**0**	**0**	**0**
	**4.**	**0**	**0**	**0**	**0**	**0**	**0**	**0**
	**5.IFN**	**0**	**0**	**0**	**0**	**0**	**0**	**0**
**IL-37**	**1.**	**0**	**-**	**-**	**-**	**0.9**	**1**	**0.2**
	**2.**	**0**	**-**	**-**	**-**	**0.3**	**0.5**	**0.6**
	**3.**	**0**	**0**	**0**	**0.3**	**5**	**0.7**	**0.6**
	**4.**	**0**	**0**	**0**	**0.1**	**2**	**0.1**	**0.4**
	**5.IFN**	**0**	**0**	**0**	**0**	**0**	**0**	**0**
**CCL11**	**1.**	**0**	**-**	**-**	**-**	**0**	**0**	**0.3**
	**2.**	**0**	**-**	**-**	**-**	**0.1**	**0**	**0**
	**3.**	**0**	**0**	**0**	**0**	**0**	**0**	**0.2**
	**4.**	**0**	**0**	**0**	**0**	**0**	**0.1**	**0.2**
	**5.IFN**	**0**	**0**	**0**	**0**	**0**	**0**	**0**
**CCL16**	**1.**	**0**	**-**	**-**	**-**	**0.1**	**0.2**	**0**
	**2.**	**0**	**-**	**-**	**-**	**0**	**0**	**0**
	**3.**	**0**	**0**	**0**	**0**	**0.2**	**0**	**0**
	**4.**	**0**	**0**	**0**	**0**	**0**	**0**	**0**
	**5.IFN**	**0**	**0**	**0**	**0**	**0**	**0**	**0**
**CCL17**	**1.**	**0**	**-**	**-**	**-**	**0**	**1**	**3**
	**2.**	**0**	**-**	**-**	**-**	**30**	**79**	**95**
	**3.**	**0**	**0**	**0**	**0**	**0**	**0**	**2**
	**4.**	**0**	**0**	**0**	**0**	**0**	**2**	**4**
	**5.IFN**	**0**	**0**	**0**	**0**	**0**	**0**	**0**
**CCL25**	**1.**	**0**	**-**	**-**	**-**	**0**	**0**	**0.7**
	**2.**	**0**	**-**	**-**	**-**	**0**	**0**	**0**
	**3.**	**0**	**0**	**0**	**0**	**0**	**0**	**0**
	**4.**	**0**	**0**	**0**	**0**	**0**	**0**	**0**
	**5.IFN**	**0**	**0**	**0**	**0**	**0.2**	**0.1**	**0.4**
**CCL26**	**1.**	**0.2**	**-**	**-**	**-**	**0.3**	**0**	**9**
	**2.**	**0**	**-**	**-**	**-**	**0.3**	**0**	**0.2**
	**3.**	**0**	**0**	**0**	**0**	**0**	**0**	**0**
	**4.**	**0**	**0**	**0**	**0**	**0**	**0**	**0**
	**5.IFN**	**0**	**0**	**0**	**0**	**0**	**0**	**0**

**CXCL9**	**1.**	**2**	**-**	**-**	**-**	**0.3**	**0**	**0.5**	
	**2.**	**3**	**-**	**-**	**-**	**0.3**	**0.3**	**0.3**	
	**3.**	**0.2**	**0**	**0**	**2**	**0**	**0**	**1**	
	**4.**	**2**	**0.2**	**0**	**4**	**0**	**0**	**0**	
	**5.IFN**	**3**	**1**	**1**	**1**	**633**	**6084**	**4671**	**100x (2000x)**
**CXCL12**	**1.**	**0**	**-**	**-**	**-**	**0**	**0**	**0**	
	**2.**	**0**	**-**	**-**	**-**	**0**	**0**	**0**	
	**3.**	**0**	**0**	**0**	**0**	**0**	**0**	**0**	
	**4.**	**0**	**0**	**0**	**0**	**0.1**	**0**	**0**	
	**5.IFN**	**0**	**0**	**0**	**0**	**0**	**0.1**	**0**	
**CXCL13**	**1.**	**0**	**-**	**-**	**-**	**0.1**	**1**	**3**	
	**2.**	**2**	**-**	**-**	**-**	**0.6**	**2**	**1**	
	**3.**	**0.5**	**0**	**0.6**	**0**	**0**	**27**	**42**	
	**4.**	**0**	**0**	**0**	**0**	**0**	**2**	**4**	
	**5.IFN**	**0**	**0**	**0**	**0**	**0**	**0**	**0**	
**CXCL14**	**1.**	**0**	**-**	**-**	**-**	**2**	**0.5**	**0.2**	
	**2.**	**0**	**-**	**-**	**-**	**0.6**	**0.1**	**0.1**	
	**3.**	**0**	**0.5**	**0.2**	**0**	**0.4**	**0**	**0**	
	**4.**	**0**	**1**	**0**	**0**	**2**	**0.7**	**0.5**	
	**5.IFN**	**0**	**0.4**	**0**	**0**	**0.2**	**0**	**0**	
**CXCL17**	**1.**	**0**	**-**	**-**	**-**	**0**	**0.2**	**0**	
	**2.**	**0**	**-**	**-**	**-**	**0**	**0**	**0**	
	**3.**	**0**	**0**	**0**	**0**	**0**	**0**	**0**	
	**4.**	**0**	**0**	**0**	**0**	**0**	**0**	**0**	
	**5.IFN**	**0**	**0**	**0**	**0**	**0**	**0**	**0**	
